# Self-Healing Hydrogel Membrane Provides a Strategy for the Steady Production of Clean Water from Organic Wastewater

**DOI:** 10.3390/membranes13070648

**Published:** 2023-07-05

**Authors:** Xin Li, Jionghao Feng, Haijun Wang, Florian Ion Tiberiu Petrescu, Ying Li

**Affiliations:** 1 The Key Laboratory of Food Colloids and Biotechnology, School of Chemical and Material Engineering, Jiangnan University, Wuxi 214122, China; lixin951001@163.com (X.L.); 1052200311@stu.jiangnan.edu.cn (J.F.); wanghjzj@163.com (H.W.); 2 Department of Mechanisms and Robots Theory, Bucharest Polytechnic University, 060042 Bucharest, Romania

**Keywords:** hydrogel membrane, photothermal, photocatalytic, self-healing, hydrogel, light trapping

## Abstract

When the typical solar-driven hydrogel water evaporator treats the organic sewage, the organic pollutants will be accumulated in the evaporator and affect the evaporation performance. This issue is resolved by using silver–disulfide bonding to fix the silver oxide/silver (Ag_2_O/Ag) nanoparticles inside the polyacrylamide-acrylic acid hydrogel, resulting in the photocatalytic degradation of methyl orange and solar-driven water evaporation. Ag_2_O/Ag nanoparticles are a solar–thermal conversion material used to replace the traditional carbon material. On the one hand, the heterojunction structure of Ag_2_O/Ag enhances the separation ability of the photogenerated carriers, thereby increasing the photocatalytic efficiency. On the other hand, the surface of the nanoparticles is grafted with N, N′-bis(acryloyl) cystamine and becomes the crosslinking agent which is fixed in the hydrogel. Meanwhile, the inverted pyramid structure can be built at the surface of the hydrogel by soft imprinting technology. This kind of structure has excellent light trapping performance, which can increase the efficiency of Ag_2_O/Ag photocatalysis. Furthermore, the dynamic reversible coordination effect between Fe^3+^ and carboxyl realizes the self-healing capability of the hydrogel. Here are the properties of hydrogel: the fracture stress is 0.35 MPa, the fracture elongation is 1320%, the evaporation rate is 1.2 kg·m^−2^·h^−1^, and the rate of the photocatalytic degradation of methyl orange is 96% in 3 h. This self-healing hydrogel membrane provides a strategy to steadily get clean water from organic sewage.

## 1. Introduction

With the rapid growth of the world population, the demand for fresh water has been increasing year by year. With readily available fresh water in short supply, desalination and contaminated water purification have come under the spotlight as alternative sources of clean water [1]. Compared to traditional seawater purification technologies, such as reverse osmosis and ion exchange, some novel distillation systems show potential for evaporation-based water purification due to high efficiency, low cost, and scalability. In this context, exploring sustainable solar energy to power water evaporation has become a rapidly growing research direction [2,3]. There is a great deal of interest in improving the efficiency of solar desalination, and the key design principles can be broadly summarized as follows: (I) enhancing solar absorption to collect energy from concentrated sunlight and (II) locating heat near the surface of water evaporation to concentrate solar energy to improve energy efficiency. For example, the absorption of a wide solar spectrum is enhanced with various absorbers with isoplasmons or narrow band gaps to further improve the efficiency of solar energy collection, such as graphene [4], polypyrrole (PPy) [5], and plasma nanoparticles [6]. In addition to improving light absorption, the material design also locates heat (from solar energy conversion) on the surface of water evaporation to inhibit heat loss and improve energy efficiency. For example, a Janus structure [7] is designed to reduce the low water content of the evaporation surface and reduce the energy consumption of water heating, and bionic structure is designed, such as mushroom [8], tree and leaf structure [9], to reduce the energy loss in the process of solar water evaporation.

A hydrogel is a polymer network that contains a large number of water molecules. Hydrogels can be precisely controlled through the customization of crosslink points, change of monomer molecules, and other material modifications to obtain the desired chemical and physical properties [10]. There are a large number of pores in the hydrogel, which can be used as a channel for water transmission. At the same time, the molecular chains in the hydrogel have a strong hydrogen bond between water molecules, which can reduce the enthalpy of evaporation to achieve a high evaporation rate [11]. For example, Zhou et al. [12] used reduced graphene oxide as a photothermal conversion material to prepare polyvinyl alcohol (PVA) system, which can achieve a high evaporation rate (~2.5 kg·m^−2^·h^−1^) under 1 sun irradiation (1 kW·m^−2^). PVA significantly encourages water evaporation by reducing the enthalpy of water evaporation in the hydrogel network. For example, Chen et al. [13] constructed a novel Janus solar evaporator by loading Ppy nanobelts onto a PVA hydrogel, which can achieve an evaporation rate of 2.64 kg·m^−2^·h^−1^. The Janus evaporator also has excellent salt-resistant desalination and contaminant purification performance in seawater and sewage. However, the presence of organic pollutants will form a shielding coating covering the photo thermal film and reduce the photo thermal conversion efficiency of the solar evaporator [14,15]. Therefore, it is urgent to develop solar evaporators with the degradation of organic matter in polluted water to meet the needs of photohot water evaporation.

Based on the above problems, a kind of self-healing hydrogel evaporator was designed with the integrated photocatalyst/photothermal agent and uniform dispersion property. A hydrogel monomer was synthesized using acrylamide (AAm) and acrylic acid (AA) as the monomers, N, N′-methylene bisacrylamide (MBAAm) as the covalent crosslinking agent, an iron ion provided by ferric nitrate as the dynamically reversible coordination crosslinking agent, and ammonium persulphate as the initiator. At the same time, the surface of Ag_2_O/Ag nanoparticles is modified with N, N′-bis(acryloyl) cystamine (BACM) as the covalent crosslinking agent to react with the hydrogel and disperse homogeneously in the evaporators. Ag_2_O/Ag nanoparticles realize the synergies of the photocatalytic degradation of methyl orange and photothermal water evaporation. A silver–disulfide bond provides the hydrogel evaporators with a self-healing property.

## 2. Materials and Methods

### 2.1. Chemicals and Materials

Silver nitrate, ferric nitrate nonahydrate, polyvinylpyrrolidone, potassium hydroxide, ethanol, AAm, AA, MBAAm, BACM, ammonium persulphate, and methyl orange (MO) were purchased from Sigma Aldrich Trading Co., Ltd. (Shanghai, China). All reagents were used directly without further purification. Monocrystalline silicon was purchased from GRINM Semiconductor Materials Co., Ltd. (Beijing, China).

### 2.2. Preparation of Silver Oxide/Silver Nanoparticles

Silver nitrate was dissolved in deionized water to prepare a 0.2 mol/L silver nitrate solution. Polyvinylpyrrolidone weighing twice as much as silver nitrate was added in above solution and stirred for 30 min to dissolve polyvinylpyrrolidone. Then 1 mol/L of potassium hydroxide was added to the solution. The precipitate obtained with liquid centrifugation was washed to pH 7 and dried at low temperature to obtain silver oxide (Ag_2_O) nanoparticles. The Ag_2_O nanoparticles were dispersed in ethanol and then irradiated by 30 W UV lamp for 60 min to reduce part of Ag_2_O. Then, the nanoparticles were dried at 70 °C for 1 h to obtain the dry Ag_2_O/Ag nanoparticles.

### 2.3. Preparation of Hydrogel

AAm (2 g), AA (300 μL), and MBAAm (10 mg) were dissolved in 10 mL deionized water. Different masses of ferric nitrate (6 mg, 12 mg, 18 mg, 24 mg, 30 mg) and ammonium persulfate (4 mg) were added to the above solution to obtain the precursor solution. Hydrogel was prepared by curing the precursor solution at a certain temperature (60 °C, 70 °C, 80 °C, 90 °C, 100 °C) for a certain time (1 h, 2 h, 3 h, 4 h, 5 h).

### 2.4. Preparation of Solar-Driven Catalyst and Water Evaporation Hydrogel Membrane

Based on the anisotropic etching of the monocrystalline silicon in the alkaline environment, a monocrystalline silicon with pyramid structure was obtained as a curing template after etching for 30 min. Ag_2_O/Ag nanoparticles (0.5 mg, 1.5 mg, 2.5 mg, 3.5 mg, 4.5 mg) are mixed with BACA as the mass ratio 2:1 and dispersed in the deionized water ultrasonically. Then, 2 g AM, 300 μL AA, and 10 mg MBAA were dissolved in the liquid. After the ferric nitrate nonahydrate was dissolved, 4 mg ammonium persulfate was added as initiator to get precursor dispersion. The precursor dispersion was put into the oven at a certain temperature to get the hydrogel.

### 2.5. Characterization

The morphology of hydrogel was observed with a scanning electron microscope (SEM, Hitachi S-4800, Tokyo, Japan). The hydrogel should be freeze dried and magnetron sputtered with the nanogold conductive layer ahead of time. UV-vis spectra were recorded using a UV-vis spectrometer (UV-3600 plus, Shimadzu, Kyoto, Japan). The X-ray diffraction (XRD) pattern of the sample was measured with a Bruker D2 PHASER X-ray diffractometer (D2, Bruker, Berlin, Germany) equipped with Cu-Ka radiation (λ = 1.540, 598 Å). The molecular structures of the samples were characterized using a Fourier Transform Infrared Spectrometer (FTIR) (Nicolet 6700, Thermo Fisher Scientific, Waltham, MA, USA). The mechanical strength of the hydrogel was characterized using a double-column bench test system (5967X, ITW, Cincinnati, OH, USA) at a stretching speed of 200 mm·min^−1^. The rheological performance of the samples was characterized using a rotational rheometer (DHR-3, TA, New Castle, DE, USA), including oscillation–amplitude sweep, oscillation–frequency sweep, and oscillation–time sweep. Water evaporation, photothermal conversion and photocatalytic performance were carried out under a 300 W xenon lamp (CEL-HXF 300, CEAuLight Co., Ltd., Beijing, China) as the visible light source. The weight change of water evaporation occurred every 5 min for 30 min and the temperature change of hydrogel membrane every 1 min for 30 min. The hydrogel was put into the MO solution (5 mg·L^−1^, 5 mL) in the dark environment for 30 min. Then, the residual MO concentration was recorded every 0.5 h for 4 h under illumination. 

## 3. Results and Discussion

### 3.1. Preparation Strategy of Solar-Driven Catalyst and Water Evaporation Hydrogel Membrane

**Figure 1:**
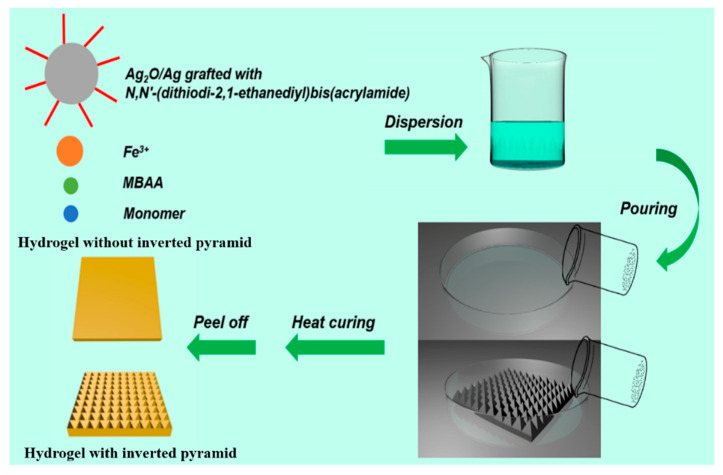
Preparation process of solar-driven catalyst and water evaporation hydrogel membrane.

At first, the monocrystalline silicon with a pyramid structure as a hydrogel curing template was prepared with anisotropic etching. Figure 1 shows the preparation process of a solar-driven catalyst and the water evaporation hydrogel membrane with an inverted pyramid structure. AAm and AA as the copolymerzation monomers, MBAAm as the covalent crosslinking agent, and ferric nitrate nonahydrate as the coordination crosslinking agent were dissolved in deionized water. Ammonium persulphate was utilized as the thermal initiator and subsequently poured into a suitable mold, such as a planner or inverted pyramid mold, for heat curing in order to obtain a pure hydrogel. At the same time, the hydrogel precursor mixed with Ag_2_O/Ag nanoparticles grafted by BACM was also poured into a suitable mold to obtain the hydrogel filled with Ag_2_O/Ag nanoparticles. Both kinds of hydrogels have an inverted pyramid structure if the selected mold has the inverted pyramid structure.

**Figure 2:**
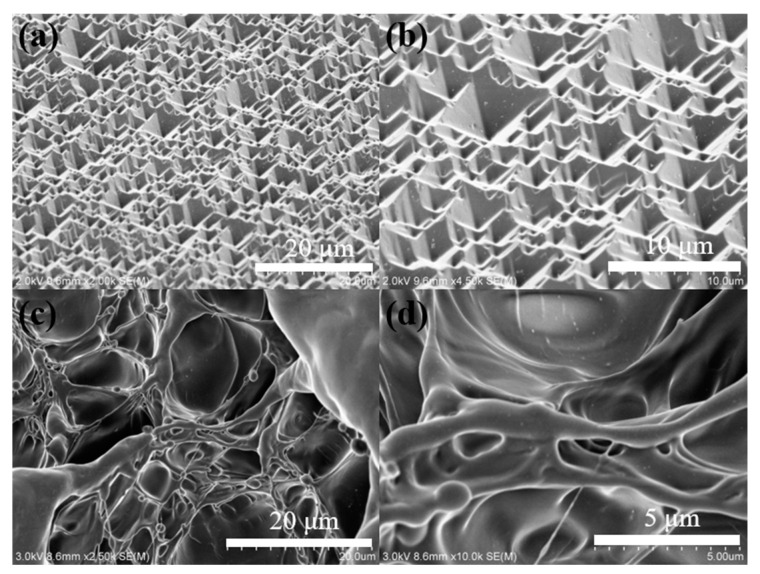
SEM image: (**a**,**b**) the inverted pyramid structure on the surface of the solar-driven catalyst and water evaporation hydrogel membrane; (**c**,**d**) the porous network structure of the hydrogel.

Figure 2 shows the SEM images of the processed hydrogel. As shown in Figure 2a,b, the hydrogel after soft lithography exhibits an inverted pyramid structure that is shape-complementary to the silicon template. The uneven surface structure can improve its light trapping ability, which means the incident light can reflect in the inverted pyramid cavity several times and be absorbed adequately. This kind of structure increases the surface area and improves the evaporation rate. Figure 2c,d show the porous network structure of the hydrogel by observing the liquid nitrogen brittle fracture cross-section. A wealth of pores provides a way to transfer water. The size of the pores (>1 μm) is larger than the molecular size of methyl orange (<2 nm) [16]. Methyl orange can easily penetrate the hydrogel and come into contact with silver/silver oxide nanoparticles, thereby enabling solar-driven catalysis in the hydrogel.

**Figure 3:**
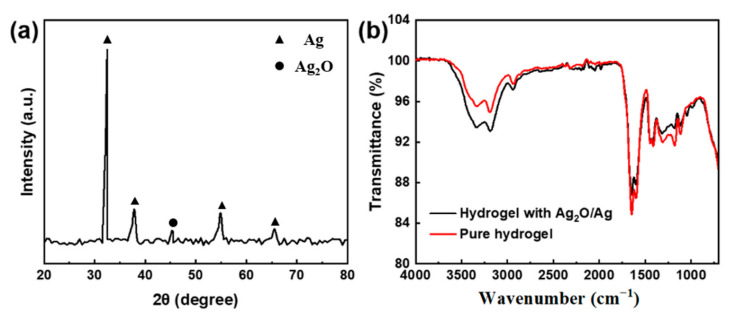
Characterization of the composition of Ag_2_O/Ag nanoparticles and the hydrogels: (**a**) XRD spectrum of Ag_2_O/Ag nanoparticles; (**b**) FTIR spectrum of the hydrogels.

The compositions of Ag_2_O/Ag nanoparticles were characterized by XRD, which confirmed the successful synthesis of the Ag_2_O/Ag nanoparticles. Figure 3a shows the XRD spectrum of Ag_2_O/Ag nanoparticles. The presence of silver is confirmed by the peaks at 32°, 38°, 55°, and 65°. The presence of Ag_2_O is confirmed by the peak at 45° [17]. This spectrum means that Ag_2_O/Ag nanoparticles are prepared successfully. The hydrogel was characterized by IR, and it was confirmed that the structure of the hydrogel would not be damaged when Ag_2_O/Ag nanoparticles were added to the hydrogel. Figure 3b shows the FTIR spectrum of the hydrogel. The absorption peak at 3323 cm^−1^ corresponds to the stretching vibration of NH_2_ of polyacrylamide (PAAm). The absorption peak at 3187 cm^−1^ corresponds to the stretching vibration of the O-H of poly acrylic acid (PAA). The absorption peak at 2931 cm^−1^ corresponds to the stretching vibration of C-H of molecular chain. The absorption peaks at 1645 cm^−1^ and 1600 cm^−1^ correspond to the stretching vibration of C=O of PAAm [18,19]. The absorption peak at 1315 cm^−1^ means the coordination bond is formed between Fe^3+^ and the carboxyl of PAA. The absorption peak at 1417 cm^−1^ corresponds to the stretching vibration of C-N. All of these means the hydrogel is prepared successfully.

### 3.2. Tensile Properties

**Figure 4:**
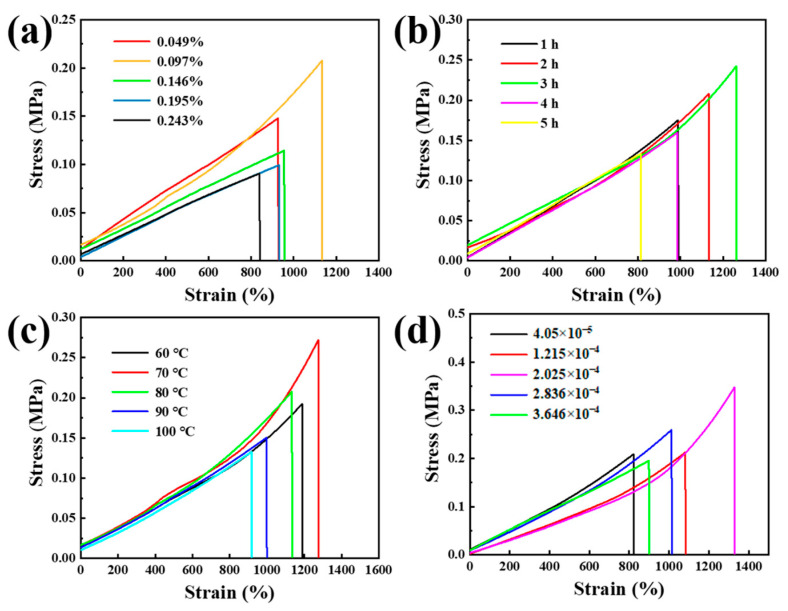
The stress–strain curve of the hydrogel with different curing conditions: (**a**) Fe^3+^ addition, (**b**) curing temperature, (**c**) curing time, and (**d**) adding amount of Ag_2_O/Ag.

The mechanical properties of the hydrogels play a key role in the practical application. The mechanical properties of hydrogels are mainly affected by crosslinking density, molecular weight, filler, and water content [10]. In this study, the influence of the Fe^3+^ dosage, curing temperature and curing time on the mechanical properties of hydrogels was investigated. Figure 4 is the stress–strain curves of the dumb-bell shaped hydrogels. Figure 4a shows the effect of the Fe^3+^ dosage on the hydrogel. The mass dosage of ferric chloride is converted into a mass percent. For example, a dosage of 6 mg ferric chloride corresponds to a concentration of 0.049%. It is evident that the tensile properties of the hydrogel initially increase and subsequently decrease with increasing dosage. When the ferric nitrate nonahydrate mass percent is 0.097%, the hydrogel shows the highest tensile properties. In such a condition, the fracture elongation goes up to 1150%, and the fracture stress goes up to 0.22 MPa. When the dosage of Fe^3+^ is low, the hydrogel exhibits poor tensile properties. However, as the content of Fe^3+^ increases, the crosslinking density also increases, and the polymer network become stronger. As a result, there is a significant improvement in the tensile properties, but as the catalyst for PAAm chemical degradation, Fe^3+^ will reduce molecular weight of hydrogel thereby weakening the tensile properties if the dosage is too much [20,21]. The following study is based on the result that the ferric nitrate nonahydrate mass percent is 0.097%. Figure 4b shows the effect of curing time on the tensile properties when the curing temperature is 70 °C. Extending the curing time improves the tensile properties by the way of increasing the percent conversion of the polymerization. However, extending the curing time will reduce the water content of the hydrogel after the monomers are used up. Therefore, the tensile properties of the hydrogel are best when the curing time is 3 h. In this condition, the fracture elongation goes up to 1250%, and the fracture stress goes up to 0.25 MPa. Figure 4c shows the effect of curing temperature on tensile properties when the Fe^3+^ dosage and curing time are fixed. The tensile properties increase firstly and decrease soon with the increase of curing temperature. The reason is that the increasing curing temperature can increase molecular weight and improve the tensile properties, but the excessive temperature will reduce the tacticity of the polymer and weaken the tensile properties. The tensile properties are the best when the curing temperature is 70 °C. The fracture elongation goes up to 1280%, and the fracture stress goes up to 0.27 MPa at 70 °C. Based on the best mechanical strength, Ag_2_O/Ag nanoparticles grafted with BACM was added to realize the solar-driven catalyzing methyl orange degradation and the water evaporation. Figure 4d shows the effect of Ag_2_O/Ag nanoparticles’ adding amount on the tensile properties. The adding amount is converted into a mass content. For example, 4.05 × 10^−5^ means the proportion between the mass of Ag_2_O/Ag nanoparticles and the hydrogel is 4.05 × 10^−5^. The different proportion exhibits different tensile properties, and when the proportion is 2.025 × 10^−4^, the hydrogel shows the best tensile property. The fracture elongation goes up to 1320%, and the fracture stress goes up to 0.35 MPa. The hydrogel with Ag_2_O/Ag nanoparticles shows better tensile properties than the hydrogel without Ag_2_O/Ag nanoparticles. Ag_2_O/Ag nanoparticles grafted with BACM can be used as the cross-linking agent to increase the cross-linking degree. However, the excessive addition of nanoparticles will make the tensile property of the hydrogel worse due to the poor compatibility.

### 3.3. Rheological Properties

**Figure 5:**
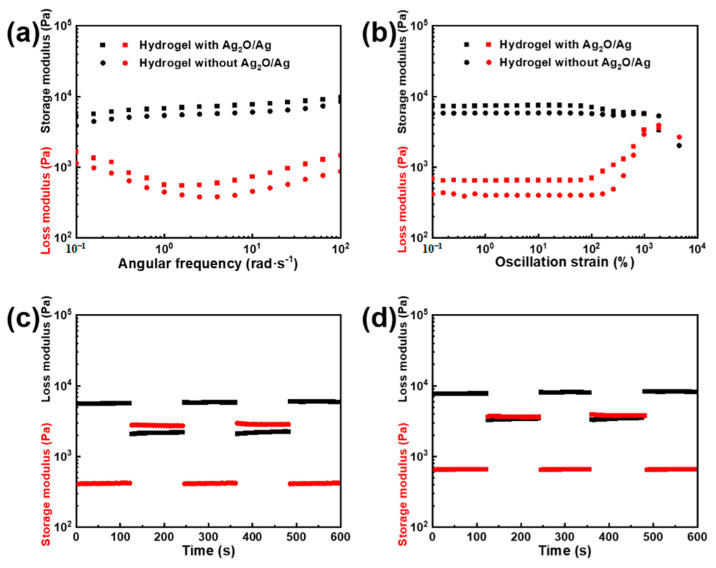
Rheological test of hydrogel, (**a**) the oscillation frequency sweep of the hydrogel, (**b**) the oscillation amplitude sweep of the hydrogel, (**c**,**d**) the continuous step oscillation amplitude sweep of the hydrogel, each stage lasts 120 s.

The rheological properties of the hydrogel play a crucial role in practical applications. The rheological properties of the hydrogel are influenced by Ag_2_O/Ag nanoparticles, as showed in Figure 5. Figure 5a shows the oscillation frequency sweep of the hydrogel. The storage modulus is greater than the loss modulus obviously at the frequency from 0.1 to 100 rad·s^−1^ and the hydrogel with Ag_2_O/Ag nanoparticles has higher storage modulus. This indicates the hydrogel skeletons network have high strength and the hydrogel shows obvious solid property. Ag_2_O/Ag grafted with BACM increases the hydrogel crosslinking density, as the cross-linking agent. Figure 5b shows the oscillation frequency sweep of the hydrogel. With the oscillation strain increasing, the hydrogel keeps solid stably at first. When the oscillation strain goes up to 100%, the storage modulus decreases, the loss modulus increases, and the hydrogel shows viscosity. When the oscillation strain goes up to 200%, the loss modulus is higher than the storage modulus regardless of whether the hydrogel contains Ag_2_O/Ag nanoparticles or not, meaning the hydrogel skeleton networks are broken. Figure 5c,d show the continuous step oscillation–amplitude sweep of the hydrogel; each stage lasts 120 s. To verify the hydrogel self-healing capability, low strain (1%) and high strain (1200%) are carried out on the hydrogel alternately. These figures show how the storage modulus and loss modulus change in this process. In the first stage, the storage modulus is always higher than the loss modulus when there is only small-scale oscillatory shearing. This means that the network structure is undamaged. In the second stage, with the high strain being caused, the storage modulus declines sharply and is lower than the loss modulus, meaning that the network structure is broken. Then, in the third stage, with the use of low strain to replace high strain, the storage modulus and loss modulus recover the initial values quickly and remain stable. This indicates that the hydrogel has an excellent self-healing capability to repair its network structure quickly. In the next circulations, the results are similar as the above one. This indicates that the hydrogel self-healing capability is repeatable. An excellent repeatable self-healing capability enables the hydrogel to maintain stability even under severe external disturbances for extended periods of time.

### 3.4. Optical and Photothermal Properties

**Figure 6:**
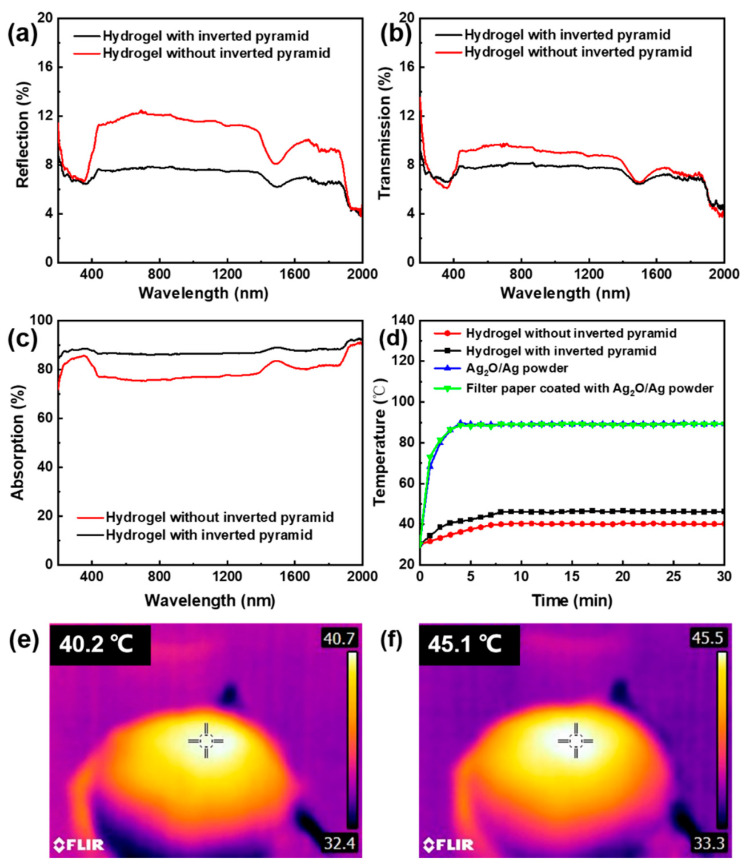
Comparison of light absorption capacity of hydrogel with and without inverted pyramid structure: (**a**) reflectance, (**b**) transmittance, (**c**) absorbance, (**d**) photothermal temperature of different materials, (**e**,**f**) the temperature image of the surface of the hydrogel after being irradiated for 30 min under 1 solar power density. The cross in each subfigure represents the temperature measured at this point.

The ability of light-absorption is the key factor which affects the properties of hydrogel membrane solar-driven catalytic and water evaporation. Figure 6 shows the test about the light-absorption of the hydrogel membrane and the heating property of a series of materials, which is used to analyze the rational utilization of the light energy. Figure 6a,b show the hydrogel with an inverted pyramid structure has a lower reflectance and transmittance than the hydrogel without an inverted pyramid structure. Figure 6c shows the hydrogel with an inverted pyramid also has a higher absorption ability. The reason is that the inverted pyramid structure can make the incident light reflect several times and absorb the optical energy efficiently. Figure 6d shows the materials’ temperature change with the time going by under 1 solar power density. The materials include Ag_2_O/Ag nanoparticles, the filter paper covered by Ag_2_O/Ag nanoparticles, the hydrogel without an inverted pyramid structure, and the hydrogel with an inverted pyramid structure. According to the curves, the Ag_2_O/Ag nanoparticles’ temperature goes up to 90 °C under illumination. The same phenomenon occurs in the filter paper covered by Ag_2_O/Ag nanoparticles. For the nanoparticles filled in the hydrogel, the heat is transferred to the water in the hydrogel, which cannot be enriched. The highest temperature of hydrogel surface is just 40.2 °C, as shown in Figure 6e, but for the hydrogel with the inverted pyramid structure, the temperature of the hydrogel surface can go up to 45.1 °C, as shown in Figure 6f. This is caused by the effective absorption of the incident light and reflecting the contribution of the inverted pyramid structure to improve the utilization rate of light.

### 3.5. The Photocatalytic and the Photothermal Water Evaporation Performance

**Figure 7:**
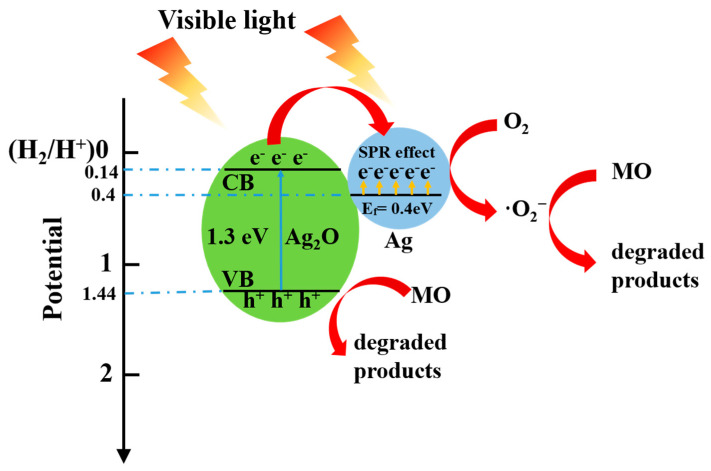
Schematic diagram of light excitation and catalytic process of Ag_2_O/Ag nanoparticles.

Silver oxide is a kind of typical photocatalyst. However, the unstable property under the illumination condition limits its applications. The Mott–Schottky heterojunction of Ag_2_O/Ag nanoparticles can accelerate the charge transfer, thus enhancing the photocatalytic ability of Ag_2_O and the photothermal conversion ability of Ag. As shown in Figure 7, Ag_2_O (Eg = 1.3 eV) [22] is excited first to produce the electron–hole pairs under the visible light irradiation. Ag_2_O/Ag can accelerate the charge transfer from the conduction band (CB) of Ag_2_O (0.14 eV vs. NHE) to Ag. This is because the Fermi energy (0.14 eV vs. NHE) of Ag is lower than the CB of Ag_2_O. The photocatalytic charges are enriched around the surface of Ag and are captured by O_2_. Then, ·O_2_^−^ is generated and then reacted with H_2_O to form ·OH, which can be used as the MO degradation. At the same time, the holes of Ag_2_O valence band (VB) can oxidize MO directly [23,24].

**Figure 8:**
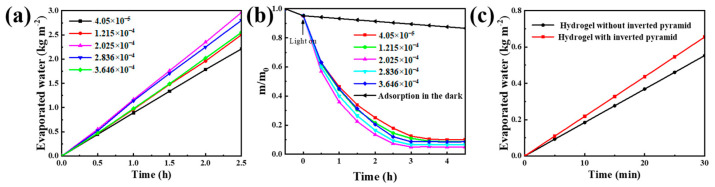
Optical function test of hydrogel: (**a**) Evaporation performance under different addition levels of Ag_2_O/Ag nanoparticles, (**b**) Photocatalytic degradation performance of MO under different addition levels of Ag_2_O/Ag nanoparticles, (**c**) Comparison of the influence of the inverted pyramid structure on the evaporation performance.

Figure 8 shows the photocatalytic and the photothermal water evaporation performance of the solar-driven catalytic synergistic water evaporation hydrogel membrane. As the photothermal conversion and photocatalytic agent, Ag_2_O/Ag nanoparticles with different addition levels can affect the photothermal water evaporation performance. Figure 8a shows the water evaporation performance of photothermal water evaporation with inverted pyramids, containing different addition levels of Ag_2_O/Ag nanoparticles. When the addition increases from 4.05 × 10^−5^ to 2.025 × 10^−4^, the evaporation rate increases and finally goes up to 1.2 kg m^−2^ h^−1^. This is because the increasing absorption and utilization of the incident light make the evaporation rate gradually increase, but if the addition is too much (addition of Ag_2_O/Ag nanoparticles >2.025 × 10^−4^), the light transmittance will be worse, and the nanoparticles at the bottom cannot absorb enough light. That is why the evaporation rate decreases totally. Figure 8b shows a similar performance when degrading MO. The photocatalytic performance of the Ag_2_O/Ag hydrogel first increases and subsequently decreases with the addition of Ag_2_O/Ag nanoparticles. When the addition is 2.025 × 10^−4^, the performance of the degradation of MO is the best, and the degradation rate can go up to 96% in 3 h. Figure 8c shows the comparison of the influence of the inverted pyramid structure on the evaporation performance. A hydrogel with an inverted pyramid (1.2 kg·m^−2^·h^−1^) has a higher water evaporation rate than a hydrogel without an inverted pyramid (1.1 kg·m^−2^·h^−1^). As above, the inverted pyramid structure can absorb the light energy more effectively. That is why the hydrogel with such a structure has a better evaporation performance. 

## 4. Conclusions

By the time that the solar-powered hydrogel water evaporator treats organic wastewater, organic pollutants will accumulate in the evaporator and affect the evaporation performance. To solve this problem, silver/silver oxide (Ag_2_O/Ag) nanoparticles are fixed inside the polyacrylamide-acrylic acid hydrogel through silver–disulfide bonds. It accomplishes the photocatalytic degradation of methyl orange and solar-driven evaporation of water. Ag_2_O/Ag nanoparticles was used as a solar-thermal conversion material to replace traditional carbon material. On the one hand, the heterojunction structure of Ag_2_O/Ag improves the separation ability of photogenerated carriers, thereby increasing the photocatalytic efficiency. On the other hand, the surface of the nanoparticles is grafted with N, N′-bis(acryloyl)cystamine and becomes the cross-linking agent that is fixed in the hydrogel. Meanwhile, the inverted pyramidal structure can be built on the hydrogel surface with soft printing technology. This kind of structure has excellent light-harvesting performance, which can increase the efficiency of Ag_2_O/Ag photocatalysis. Furthermore, the dynamic reversible coordination effect between Fe^3+^ and carboxyl realizes the self-healing ability of the hydrogel. Here are the properties of the hydrogel: the breaking stress is 0.35 MPa, the breaking elongation is 1320%, the evaporation rate is 1.2 kg·m^−2^ h^−1^, and the photocatalytic degradation rate of methyl orange is 96% in 3 h. This self-healing hydrogel membrane provides a strategy to consistently obtain clean water from organic wastewater.

In summary, the hydrogel membrane is obtained with good mechanical properties via the dynamic reversible coordination effect and chemical cross-linking. When the addition of ferric nitrate nonahydrate is 0.097%, the curing time is 3 h, the curing temperature is 70 °C, and the addition of Ag_2_O/Ag nanoparticles is 2.025 × 10^−4^, the hydrogel membrane shows great solid properties with the fracture elongation of 1320% and the fracture stress of 0.35 MPa.

The dynamic reversible coordination effect between Fe^3+^ and carboxyl provides the hydrogel membrane with a good self-healing capability. In addition, Ag_2_O/Ag nanoparticles exhibit photothermal conversion and photocatalytic performance through the link of silver–disulfide bonds. With the inverted pyramid structure, which has great light trapping performance, the hydrogel evaporation can degrade 96% of methyl orange in 3 h, and the evaporation rate can go up to 1.2 kg·m^−2^·h^−1^.

## Data Availability

Not applicable.
